# Cancer and Pancreatic Lithiasis

**Published:** 1918-09

**Authors:** 


**Cancer and Pancreatic Lithiasis.** Jean Minet, Le Prog. Med., Mch. 9, reports a case of death from pulmonary apoplexy, the liver being studded with cancerous masses varying in size from that of a pea to that of a nut, the pancreas also being cancerous and enlarged to the size of an orange, the intraglandular portion of the duct being filled with rounded mossy calculi. The cancer was of cylindro-epitheliomatous type, the calculi being pure calcium carbonate.

